# The oligomeric states of dye‐decolorizing peroxidases from *Streptomyces lividans* and their implications for mechanism of substrate oxidation

**DOI:** 10.1002/pro.5073

**Published:** 2024-06-12

**Authors:** Marina Lučić, Thomas Allport, Thomas A. Clarke, Lewis J. Williams, Michael T. Wilson, Amanda K. Chaplin, Jonathan A. R. Worrall

**Affiliations:** ^1^ School of Life Sciences University of Essex Colchester UK; ^2^ Leicester Institute for Structural and Chemical Biology, Department of Molecular and Cell Biology University of Leicester Leicester UK; ^3^ School of Biological Sciences University of East Anglia Norwich UK

**Keywords:** analytical ultracentrifugation, cryo‐EM, dodecamer, ferryl, heme, hexamer

## Abstract

A common evolutionary mechanism in biology to drive function is protein oligomerization. In prokaryotes, the symmetrical assembly of repeating protein units to form homomers is widespread, yet consideration in vitro of whether such assemblies have functional or mechanistic consequences is often overlooked. Dye‐decolorizing peroxidases (DyPs) are one such example, where their dimeric α + β barrel units can form various oligomeric states, but the oligomer influence, if any, on mechanism and function has received little attention. In this work, we have explored the oligomeric state of three DyPs found in *Streptomyces lividans*, each with very different mechanistic behaviors in their reactions with hydrogen peroxide and organic substrates. Using analytical ultracentrifugation, we reveal that except for one of the A‐type DyPs where only a single sedimenting species is detected, oligomer states ranging from homodimers to dodecamers are prevalent in solution. Using cryo‐EM on preparations of the B‐type DyP, we determined a 3.02 Å resolution structure of a hexamer assembly that corresponds to the dominant oligomeric state in solution as determined by analytical ultracentrifugation. Furthermore, cryo‐EM data detected sub‐populations of higher‐order oligomers, with one of these formed by an arrangement of two B‐type DyP hexamers to give a dodecamer assembly. Our solution and structural insights of these oligomer states provide a new framework to consider previous mechanistic studies of these DyP members and are discussed in terms of long‐range electron transfer for substrate oxidation and in the “storage” of oxidizable equivalents on the heme until a two‐electron donor is available.

## 1 INTRODUCTION

Around half of all proteins form oligomers and thus oligomerization is considered to play an essential role in the stability and function of many proteins within a cellular environment (Levy & Teichmann, 2013; Marsh & Teichmann, 2015). At its simplest, a protein oligomer can be formed from the self‐assembly of repeated copies of a single protein subunit, resulting in a homomer (Schweke et al., 2024). In contrast, a heteromer is formed from multiple distinct subunits often encoded by different genes. Structural and computational data reveal that homomers are more prevalent in prokaryotic proteomes than in eukaryotic ones, with a notable feature being that the large majority are symmetrical (Bergendahl & Marsh, 2017; Marsh & Teichmann, 2015; Schweke et al., 2024). Simulations and directed‐evolution experiments have provided convincing evidence to illustrate that symmetric assemblies, particularly homodimers, are the most energetically stable, and therefore likely to overcome the entropic costs of complex formation. Aside from an energetic advantage, what are the functional advantages of a homomeric quaternary structures? This question is not easy to answer, as advantages are often assumed without direct experimental evidence. Evolutionary studies have demonstrated that homomer distributions could be explained by stochastic, non‐adaptive processes (Lynch, 2012, 2013), and computational approaches have demonstrated that oligomerization is often a consequence arising from optimizing the thermodynamic assembly of a proteome, with no essential biological function (Jacobs et al., 2016). In the present work, we have explored the oligomerization states of the three dye‐decolorizing peroxidases (DyPs) found in the soil dwelling antibiotic producing Gram positive bacterium, *Streptomyces lividans*. Our previous work with these heme enzymes has extensively characterized their mechanism of action (Chaplin et al., 2017, 2019; Lučić et al., 2021, 2022; Lučić, Chaplin, et al., 2020; Lučić, Svistunenko, et al., 2020), revealing that distinct mechanistic differences exist between the three peroxidases. Herein, we have assessed these mechanistic differences considering the oligomerization states determined in this work.

DyPs are the most recent members of the peroxidase family to be discovered and are now known to be widely distributed in bacteria and fungi (Kim & Shoda, 1999; Lučić et al., 2021; Singh & Eltis, 2015). Structurally, DyPs comprise a dimeric α + β barrel that consists of two ferredoxin‐like folds connected by a short linker between the N‐ and C‐terminal β‐strands. The heme iron is coordinated to the polypeptide by a His residue in the C‐terminal ferredoxin‐like fold (Hofbauer et al., 2021; Sugano et al., 2007). Structure‐based sequence alignments have led to the identification of three DyP sub‐families, A and B types (bacteria) and a C/D‐type (fungi) (Ogola et al., 2009). As with other members of the peroxidase family, all three DyP sub‐families react with H_2_O_2_, to first form a two‐electron oxidized heme species called Compound I, which comprises a Fe(IV)=O species (ferryl heme) and a porphyrin π‐cation radical (Poulos, 2014). Compound I in peroxidases is highly oxidizing and reacts with an organic substrate in a one‐electron transfer step to from Compound II, where the porphyrin π‐cation radical is reduced. Compound II then oxidizes a second substrate molecule leading to the resting ferric state. Concomitant with this H_2_O_2_ induced cycle is the production of two H_2_O molecules (Poulos, 2014).

The physiological substrates of DyPs are almost completely unknown, with only a handful of examples illustrating a potential role of fungal DyPs in lignin oxidation (Liers et al., 2010, 2013) and in the degradation of an antifungal anthraquinone compound (Sugawara et al., 2019). Furthermore, the oligomeric states of DyPs have received little attention and are not well characterized and require further exploration. From the mainly structural information available, bacterial DyPs appear to be capable of forming dimeric, tetrameric, and hexameric assemblies, and fungal DyPs are exclusively monomers (Borges et al., 2022). The variation in oligomeric states among DyP members may therefore have functional consequences. A recent study has highlighted how mixtures of oligomeric states of a B‐type DyP (DtpB) from *Streptomyces coelicolor* influence the kinetic properties of substrate oxidation and thus oligomerization may serve as a mechanism to regulate catalytic activity (Pupart, Vastšjonok, et al., 2024).

Of further significance is that oligomeric states of DyPs can exist (Tang et al., 2021) when compartmentalized inside the lumen of an icosahedral hollow capsid formed by the protein encapsulin (Andreas & Giessen, 2021; Giessen & Silver, 2017; McHugh et al., 2014). Encapsulin nanoparticles can range in size from 20 to 42 nm (Giessen et al., 2019; McHugh et al., 2014; Sutter et al., 2008) and in addition to DyPs can compartmentalize other cargo proteins, such as ferritin‐like proteins (McHugh et al., 2014), proteins involved in sulfur metabolism to store crystalline elemental sulfur (Benisch et al., 2024; Nichols et al., 2021), and proteins involved in natural product synthesis (Giessen & Silver, 2017). The cargo protein is specifically targeted to the encapsulin capsid interior by a terminal localization sequence that binds to the interior face of the encapsulin (Sutter et al., 2008; Tamura et al., 2015). Several structures of intact encapsulins with their cargo proteins have been determined. These include a natively isolated encapsulin particle from *Mycobacterium smegmatis* revealing a dodecamer oligomer of DtpBs (Tang et al., 2021) and an encapsulated particle from *Haliangium ochraceum* containing a tetrahedral arrangement of ferritin decamers (Ross et al., 2022).

No gene for an encapsulin protein is encoded in *S. lividans*. The two A‐type homologs found in *S. lividans* are exported to the extracytoplasmic environment and have been named DtpA and DtpAa, whereas the DtpB, is located in the cytoplasm (Petrus et al., 2016). We have conducted sedimentation analysis using analytical ultracentrifugation (AUC) and correlated the results of the solution state assemblies obtained from the AUC experiments with interface analysis of the assembly states of the *S. lividans* DyPs found in the crystallographic asymmetric unit using the computational tool Protein Interfaces, Surfaces and Assembles (PISA) (Krissinel, 2010; Krissinel & Henrick, 2007). By using single particle cryo‐EM, we identified a hexamer assembly of DtpB to be the major component in solution, with a lower population of a dodecamer assembly also observed, illustrating that the encapsulin system for compartmentalization is not necessary to generate higher‐order assembly states of a DyP. The results are discussed considering previous mechanistic studies (Chaplin et al., 2017, 2019; Lučić et al., 2021, 2022; Lučić, Chaplin, et al., 2020; Lučić, Svistunenko, et al., 2020), in which the heme groups within these homomeric structures behave entirely independent of each other.

## 2 RESULTS

### 2.1 Oligomeric states of *S. lividans* DyPs determined by AUC

**FIGURE 1:**
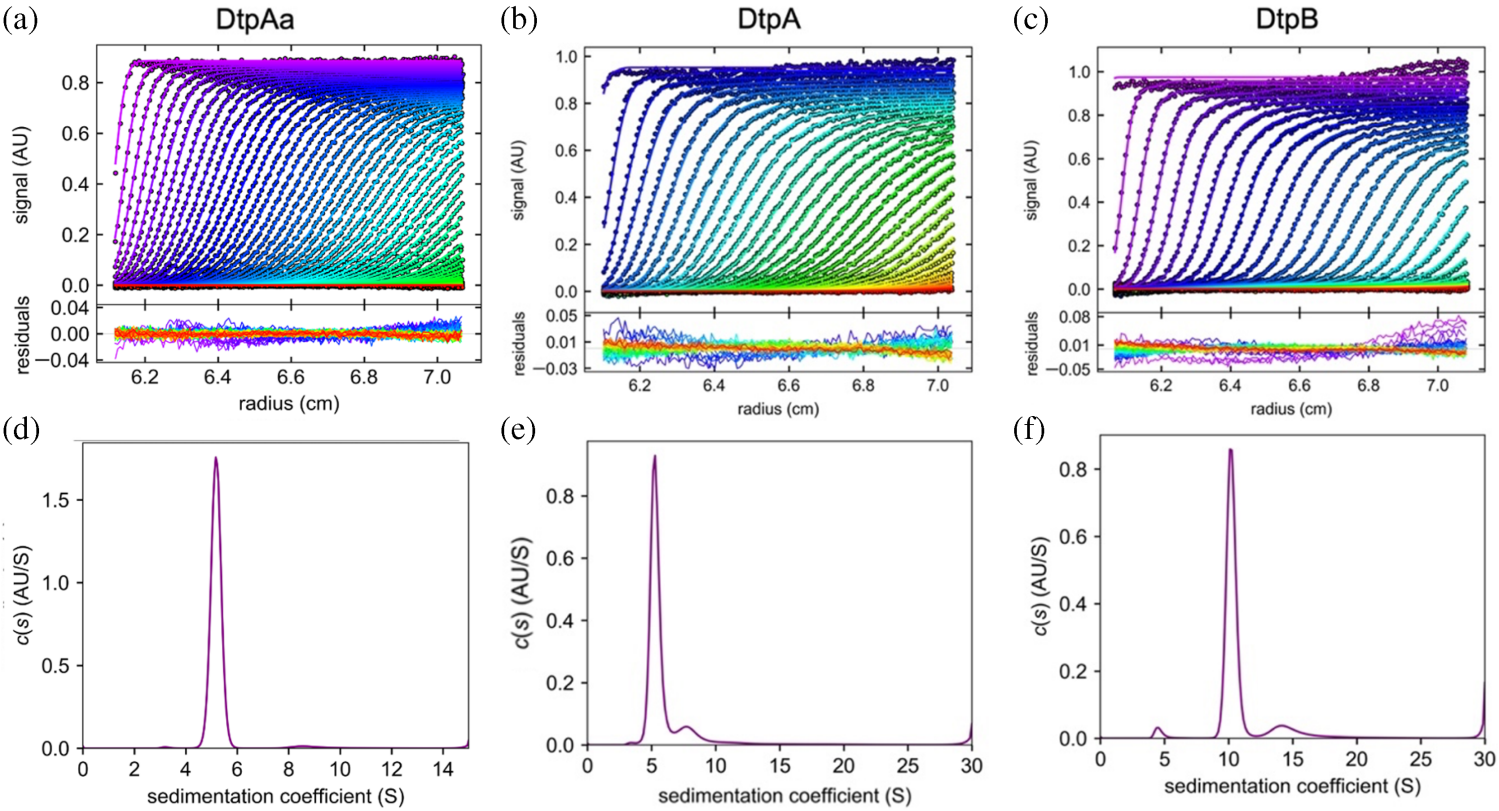
Sedimentation velocity analysis of DtpAa (a), DtpA (b), and B‐type dye‐decolorizing peroxidase (DtpB) (c) monitored by absorbance at 410 nm (points) and fitted (lines) to the Lamm equation with residual absorption from the fitted data shown below each dataset. The corresponding *c*(*S*) distribution for DtpAa (d), DtpA (e), and DtpB (f) is shown revealing the distribution of sedimenting species within each sample.

To assess the oligomeric states in solution of the three *S. lividans* DyPs, AUC was performed. AUC remains one of the most powerful techniques for the quantitative characterization of macromolecular associations and oligomeric states in solution (Howlett et al., 2006). Sedimentation velocity (SV) scans together with the distribution of sedimentation species for each of the *S. lividans* DyPs obtained from fitting of the SV data are depicted in Figure 1. For DtpAa, the *c*(*S*) distribution revealed a dominant species at 5.5 S, with some very minor components around 10 S also present (Figure 1a,d). In the case of DtpA, two separate sedimenting species were observed, a dominant species at 5.5 S, and a second minor species at 8.2 S (Figure 1b,e). Finally, for DtpB, the SV data revealed the presence of at least three sedimentation species at 4.8, 10.6, 14.9 S (Figure 1c,f). Table 1 reports the sedimentation coefficients, apparent molecular weights obtained from the *c*(*S*) distribution and the proportion (or ratio) of each sedimentation species determined for each of the DyPs from the data presented in Figure 1. The fitted frictional ratio (*f*/*f*_*0*_), a measure of a particle elongation, for DtpAa was 1.23, which is typical for a globular protein. To determine the molecular weights in the other samples, a fixed *f*/*f*_*0*_ ratio of 1.2 for each species was used. It is apparent that the dominant sedimentation species for DtpAa and DtpA are dimers, with a lower proportion of tetramer assembly present for DtpA (Table 1). For DtpB, the dominant sedimentation species has a molecular weight consistent with a hexamer (Table 1), while the two lower populated sedimented species have molecular weights indicating dimer and dodecamer oligomers, respectively, with the latter being the more populated of the two species (Table 1). We note a recent dynamic light scattering study was unable to distinguish between the nature of the oligomeric states present in a DtpB from *S. coelicolor* (Pupart, Vastšjonok, et al., 2024) as has been possible here using AUC. Therefore, not only does AUC provide definitive details of oligomer states across these DyP members but also uniquely identifies a species consistent with a dodecamer suggesting that encapsulation is not a pre‐requisite for a DyP to form a higher‐order oligomeric state.

**TABLE 1:** Summary of the analytical ultracentrifugation analysis for the three *Streptomyces lividans* DyPs.

DyP	Predicted monomer *M*_w_ (kDa)	No. of major species (ratio)	Sedimentation coefficient (S_20,W_)	Experimentally determined *M*_w_ (kDa)^a^	Assembly state and expected *M*_w_ (kDa)
DtpAa	40.3	1	5.55 ± 0.17	79.6 ± 3.7	Dimer (80.6)
DtpA	40.6	2 (76:13)	Species 1: 5.47 ± 0.43	78 ± 9	Dimer (81.2)
			Species 2: 8.26 ± 0.86	144 ± 22	Tetramer (162.4)
DtpB	34.3	3 (2.5:82:10)	Species 1: 4.76 ± 0.4	64 ± 9.4	Dimer (68.6)
			Species 2: 10.59 ± 0.4	210 ± 13	Hexamer (205.8)
			Species 3: 14.92 ± 1.0	352 ± 34	Dodecamer (411.6)

Abbreviations: DtpAa and DtpA, A‐type; FDtpB, B‐type DyP; DyP, dye‐decolorizing peroxidase.

^a^ DtpAa *M*_w_ was calculated using an experimentally fitted *f*/*f*_*0*_ ratio of 1.23. *M*_w_ values for other species were determined using a fixed *f*/*f*_*0*_ ratio of 1.2.

#### 2.1.1 *Interface analysis of the* S. lividans *DyPs*

**FIGURE 2:**
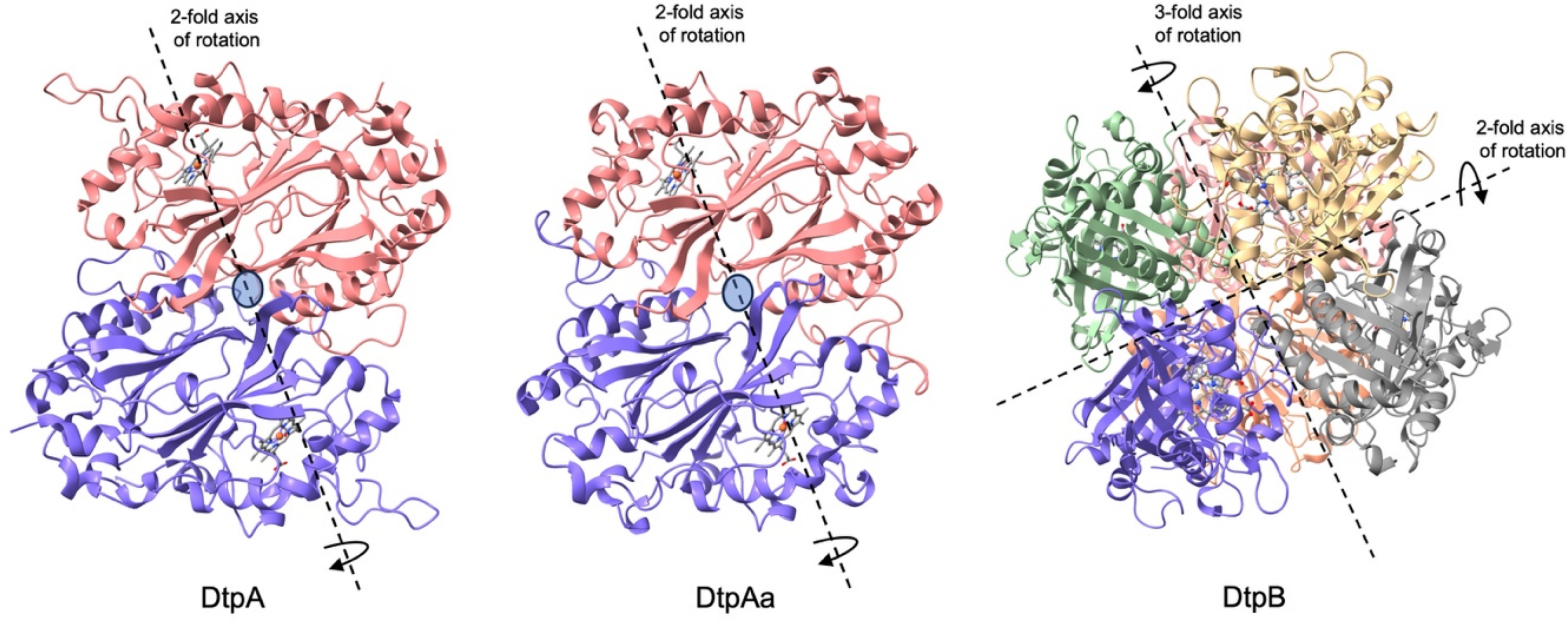
Oligomeric states found in the crystallographic asymmetric units of the three dye‐decolorizing peroxidases (DyPs) from *Streptomyces lividans*. The DtpA and DtpAa oligomers have a cyclic C2 symmetry, and the DtpB oligomer has a dihedral D3 symmetry. The rotation axis for each oligomer is indicated. PDB codes used are 6GZW (DtpA), 6TB8 (DtpAa), and 6YR4 (DtpB).

The dominant sedimentation species identified from the AUC experiments for the three *S. lividans* DyPs mirror the oligomeric states reported from previous crystallographic analysis (Chaplin et al., 2019; Lučić, Chaplin, et al., 2020; Lučić, Svistunenko, et al., 2020), depicted in Figure 2. The stability of a complex assembly in a biological system is essentially governed by the free energy of formation, solvation energy gain, interface area, hydrogen bonds, interface salt‐bridges, and hydrophobic specificity (Krissinel & Henrick, 2007). These interactions are also likely present in crystal systems (Elez et al., 2018; Gaber & Pavšič, 2021), and therefore distinguishing between crystal (non‐specific) and biological (specific) interfaces can be challenging. To assess whether the DyP crystallographic assemblies are biologically relevant and not crystal specific, an inspection of all different inter‐molecular contacts within the crystal is necessary. Various computational tools are available for this purpose (Elez et al., 2018), with PISA the most widely used (Krissinel, 2010; Krissinel & Henrick, 2007). PISA makes copies of asymmetric unit contents through the application of crystallographic symmetry operations at all possible inter‐molecular interfaces both within and between asymmetric units and then analyses interfaces and predicts complex stability.

Table 2 gives an overview of the resulting parameters obtained from the PISA analysis for the three *S. lividans* DyPs. The calculated parameters for each of the overall DyP assemblies reveal a large negative solvation free energy gain on complex formation (Δ*G* ^int^) coupled with a positive free energy of dissociation (Δ*G* ^diss^), conclusive of an assembly with high thermodynamic stability. Inspection of the PISA parameters obtained for the isologous (symmetric or head‐to‐head) interface of the A‐type DyP assemblies (Table 2) reveals DtpAa to have fewer H‐bonding interactions (N_HB_) and salt bridges (N_SB_) and a more negative solvation free energy gain (Δ*G* ^i^) upon formation of the interface compared to DtpA, implying the DtpAa homodimer possesses a more hydrophobic interface. Moreover, for both A‐type DyPs the *p*‐value of the Δ*G* ^i^ approaches zero, indicating a highly unique interaction‐specific interface, and not imposed by crystallization.

**TABLE 2:** Protein Interfaces, Surfaces and Assembles analysis of the three dye‐decolorizing peroxidases (DyPs) from *Streptomyces lividans.*

Protein (PDB code)	Overall complex				
	Surface area (Å^2^)	Buried area (Å^2^)		Δ*G* ^int^ (kcal/mol)	Δ*G* ^diss^ (kcal/mol)
DtpA (6GZW)	28,099.1	7800.2		−54.5	19.8
DtpAa (6TB8)	26,087.7	7003.9		−61.3	19.5
DtpB (6YR4)	65,410	22,840		−235.8	41.0

	Interface area (Å^2^)	Δ*G* ^i^ (kcal/mol)	Δ*G* ^i^ (*p*‐value)	N_HB_	N_SB_	CSS
Engaged interfaces DtpA and DtpAa						
DtpA	2665.3	−12.6	0.383	40	25	0.422
Chains B/A						
DtpAa	2266.9	−21.0	0.348	26	8	0.425
Chains B/A						
Isologous engaged interfaces DtpB						
Chains E/B	1148.2	−16.6	0.071	14	0	0.343
Chains C/A	1088.4	−18.3	0.032	11	5	0.343
Chains F/D	1080.9	−16.9	0.049	11	1	0.343
Heterologous engaged interfaces in order of increasing Δ*G* ^i^ for DtpB chain E						
Chains E/C	293.6	−7.2	0.049	2	0	0.091
Chains E/A	529.9	−6.6	0.288	3	5	0.164
Chains E/F	517.6	−3.9	0.489	4	5	0.164
Heterologous engaged interfaces in order of increasing Δ*G* ^i^ for DtpB chain C						
Chains C/E	293.6	−7.2	0.049	2	0	0.091
Chains C/B	509.6	−5.6	0.289	3	5	0.164
Chains C/D	557.4	−4.8	0.392	4	4	0.164
Heterologous engaged interfaces in order of increasing Δ*G* ^i^ for DtpB chain F						
Chains F/B	319.2	−8.6	0.031	1	0	0.091
Chains F/A	499.5	−5.4	0.315	4	4	0.164
Chains F/E	517.6	−3.9	0.489	4	5	0.164

Abbreviation: DtpA and DtpAa A‐type DyPs; DtpB, B‐type DyP.

In the case of the DtpB hexamer (a dimer of trimers), the PISA engaged interface analysis identifies the isologous interfaces between chains E/B, C/A, and F/D as having the largest interface area and consequently the most negative Δ*G* ^i^ values (Table 2). The *p*‐values again approach zero, indicating highly specific interfaces, as would be expected for an isologous interface. Heterologous interfaces (asymmetric or head‐to‐tail) also exist within the hexamer and are reported in Table 2. A pattern of decreasing stability based on increasing Δ*G* ^i^ values within these heterologous interfaces is observed, which coincides with an increased hydrophilic nature (increase in numbers of H‐bonds and salt bridges) of the interface and an increased interface area (Table 2). Overall, the PISA analysis supports the notion that the respective DyP complex assemblies found in the crystallographic asymmetric unit are likely to represent the dominant sedimentation species in solution (Table 1).

#### 2.1.2 Single particle cryo‐EM analysis of DtpB

**FIGURE 3:**
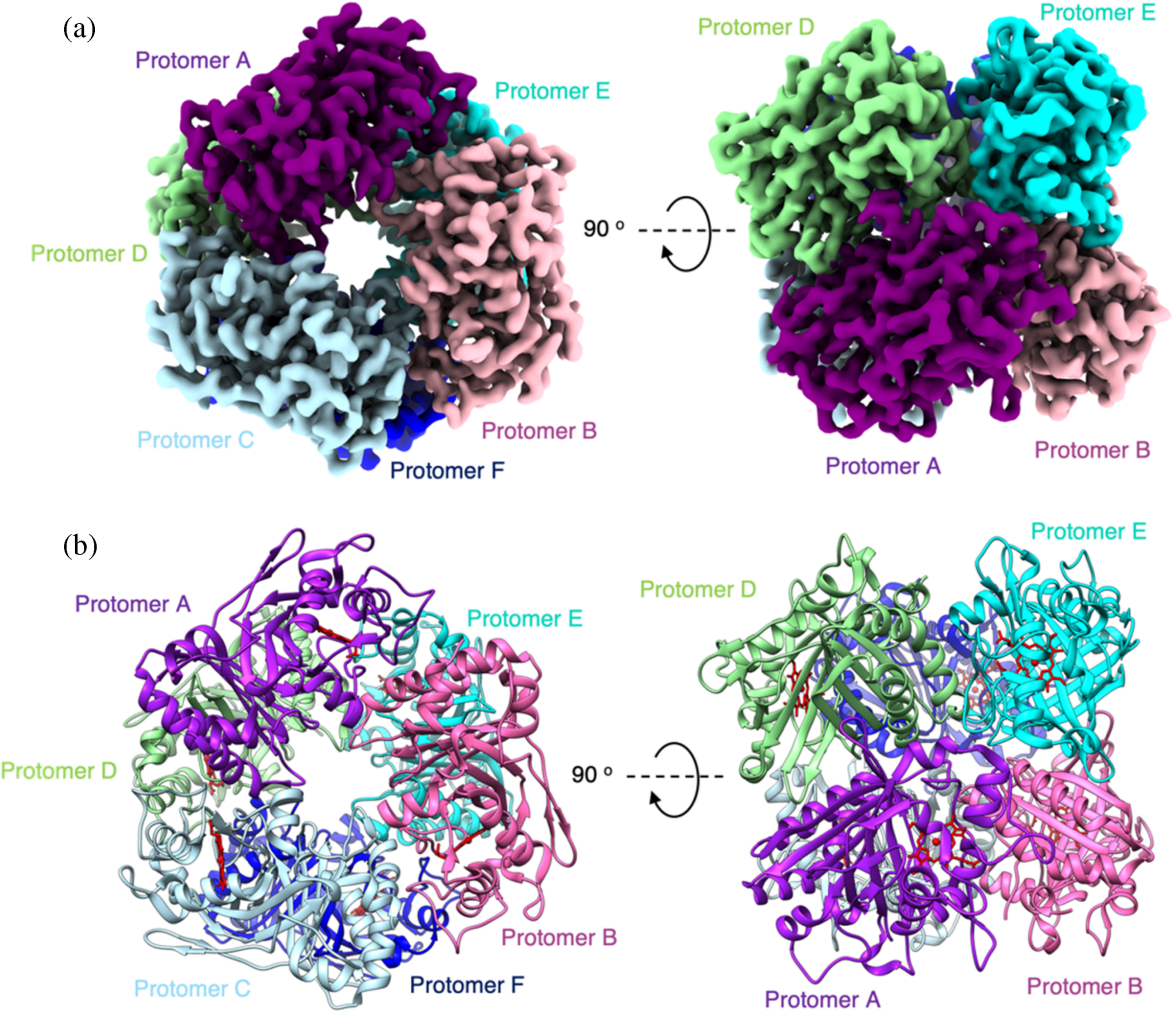
Cryo‐EM structure of the *Streptomyces lividans* DtpB hexamer. (a) Cryo‐EM maps of the DtpB hexamer assembly. On the left, the assembly viewed down the D3 symmetry axis and on the right through a 90° rotation down the C2 symmetry axis. (b) Cartoon representation of the DtpB hexamer assembly in the same orientations as in (a). Protomers colored and labeled accordingly.

We next used cryo‐EM to visualize different oligomeric states of DtpB. Prior to grid freezing a grid optimization protocol was carried out, in which the detergent CHAPSO was added to help prevent problems with preferred orientation and particle aggregation (see Section 5). Cryo‐EM data were then collected (see Supporting information and Table 3), with the angular distribution calculated in CryoSPARC (Punjani, Brubaker, et al., 2017; Punjani, Rubinstein, et al., 2017) for particle projections clearly showing that the data did not have any significant problems with preferred orientation (Figures S1 and S3). Following particle picking, hexamers of DtpB within the 2D class averages were clearly visible (Figure S1). Subsequent cryo‐EM data processing with a dihedral D3 symmetry imposed (Figure S2) resulted in a final cryo‐EM map to 3.02 Å resolution (Figure 3 and Table 3). The X‐ray structure of the ferric DtpB hexamer (Lučić, Svistunenko, et al., 2020) was used to dock within the cryo‐EM map and iterative model building and refinement conducted (Figure 3), with model quality statistics for the final model reported in Table 3. The overall assembly is compact with local resolution evenly distributed across the structure with only lower resolution observed on a few external loop regions. The clear visualization of a hexamer using single particle cryo‐EM further corroborates the conclusions from AUC data and PISA analysis that the functional state of DtpB is a hexamer.

**TABLE 3:** Cryo‐EM data collection parameters and refinement statistics for the various B‐type dye‐decolorizing peroxidase structures.

	Hexamer PDB:8RWY EMDB:19568	Quasi‐hexamer	Dodecamer
Data collection and processing			
Detector	Gatan K3	Flacon 3	
Magnification	130 k	92 K	
Energy filter slit width (eV)	20	20	
Voltage (kV)	300	200	
Flux on detector (e/pix/sec)	12.461	0.70	
Electron exposure on sample (e−/Å^2^)	47.19	40.86	
Target defocus range (μm)	0.7–2.5	1.2–2.7	
Calibrated pixel size (Å)	0.652	1.13	
Symmetry imposed	D3	C1	
Extraction box size (pixels)	520	260	
Initial particle images (no.)	71,074	43,144	
Final particle images (no.)	24,402	5324	3964
Refinement			
Map resolution at FSC = 0.143 (Å)*	3.02	14.54	13.35
Model composition			
Non‐hydrogen atoms	14,107		
Protein residues	1836		
Ligand − Heme	6		
*B* factor (Å^2^)			
Protein	99.46		
Ligand	96.58		
R.m.s deviations			
Bond lengths (Å)	0.003		
Bond angles (°)	0.485		
Validation			
Molprobity score	1.34		
Clashscore	5.29		
Poor rotamers (%)	0.00		
Ramachandran plot			
Favored (%)	97.75		
Allowed (%)	2.19		
Disallowed (%)	0.05		

**FIGURE 4:**
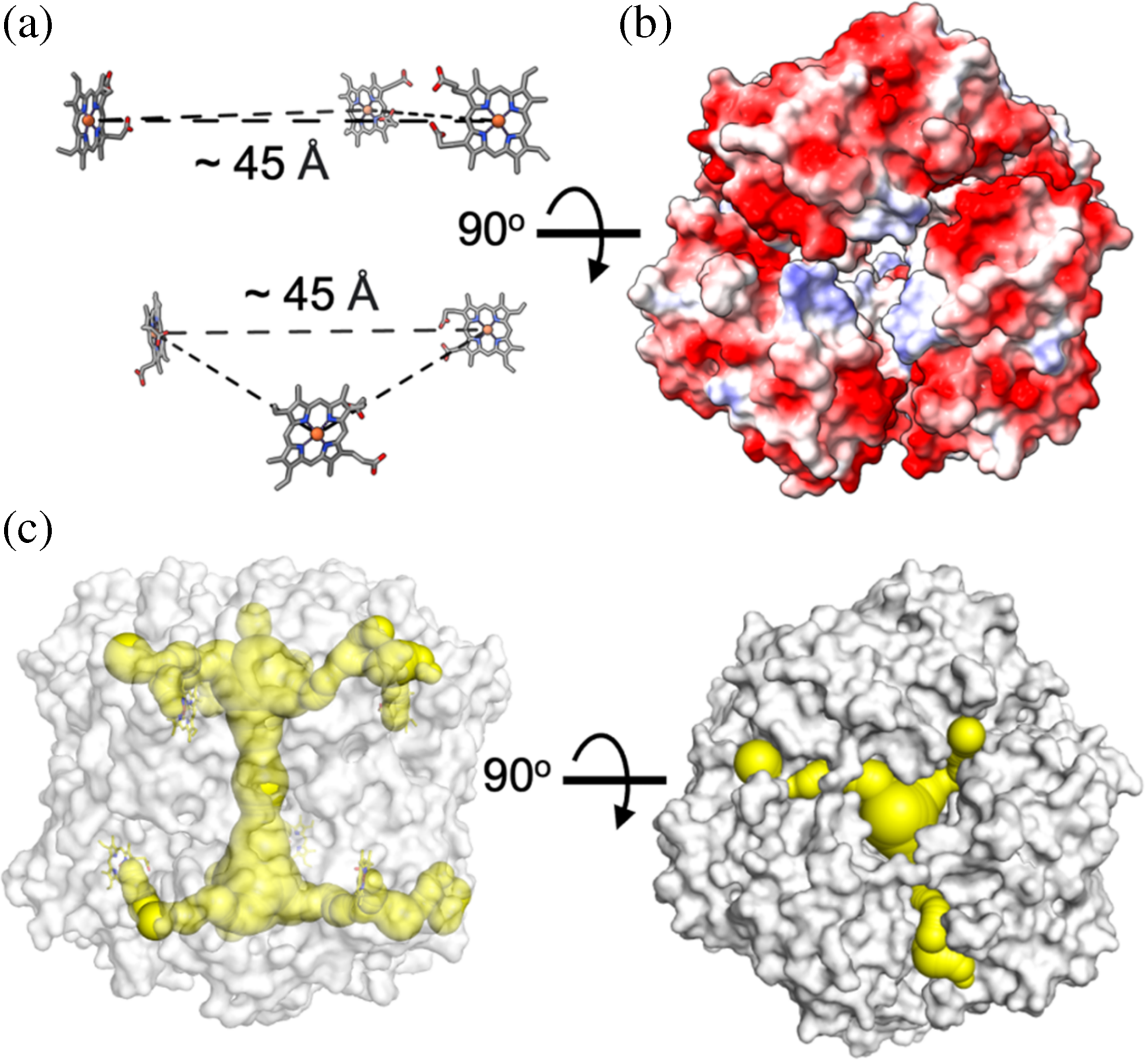
Features of the *Streptomyces lividans* DtpB B‐type dye‐decolorizing peroxidase hexamer. (a) Arrangement of heme groups within the hexamer assembly and the average iron–iron distance. (b) Electrostatic surface potential viewed looking down the D3 symmetry axis. (c) Surface representation (gray) with access channels from the heme‐iron to the surface and central pore calculated using CAVER 3.0 (Chovancova et al., 2012) shown in yellow. A probe radius of 1.2, shell depth of 8, shell radius of 7, and clustering threshold of 4 were used in the CAVER 3.0 analysis.

In each chain of the hexamer, cryo‐EM density consistent with the presence of a heme group is present (Figure S3). Within the two trimer units (chains BCD and AEF) that stack to make the dihedral hexamer assembly, the heme groups are arranged planar to each other to give iron‐to‐iron distances of 45–47 Å (Figure 4a). This distance is too long for intramolecular electron‐transfer (ET) and is indicative of the heme groups reacting in isolation. Stopped‐flow kinetic studies to monitor Compound I formation on mixing with H_2_O_2_ are indeed consistent with this structural observation (Lučić, Svistunenko, et al., 2020). The overall architecture of the cryo‐EM hexamer is dominated by a wide central channel (or pore), that makes‐up the threefold symmetry axis of the hexamer and spans it full‐length (~70 Å). The surface electrostatic map of the hexamer is dominated by negative charge, whereas the entrance to the threefold channel is lined with a ring of weak positive charge (Figure 4b). This latter feature contrasts to that of the compartmentalized DyP from *M. smegmatis*, where the entrance to the threefold channel is negatively charged (Tang et al., 2021). Further analysis of the interior of the threefold channel of DtpB reveals it to be predominately polar with CAVER 3.0 (Chovancova et al., 2012) computing access routes for H_2_O_2_ or a small substrate molecule leading from the central channel to each heme pocket and from the heme‐Fe to the hexamer surface (Figure 4c). Thus, the hexamer assembly does not occlude small molecule access to any of the heme sites.

#### 2.1.3 Visualization of a DtpB dodecamer by cryo‐EM

**FIGURE 5:**
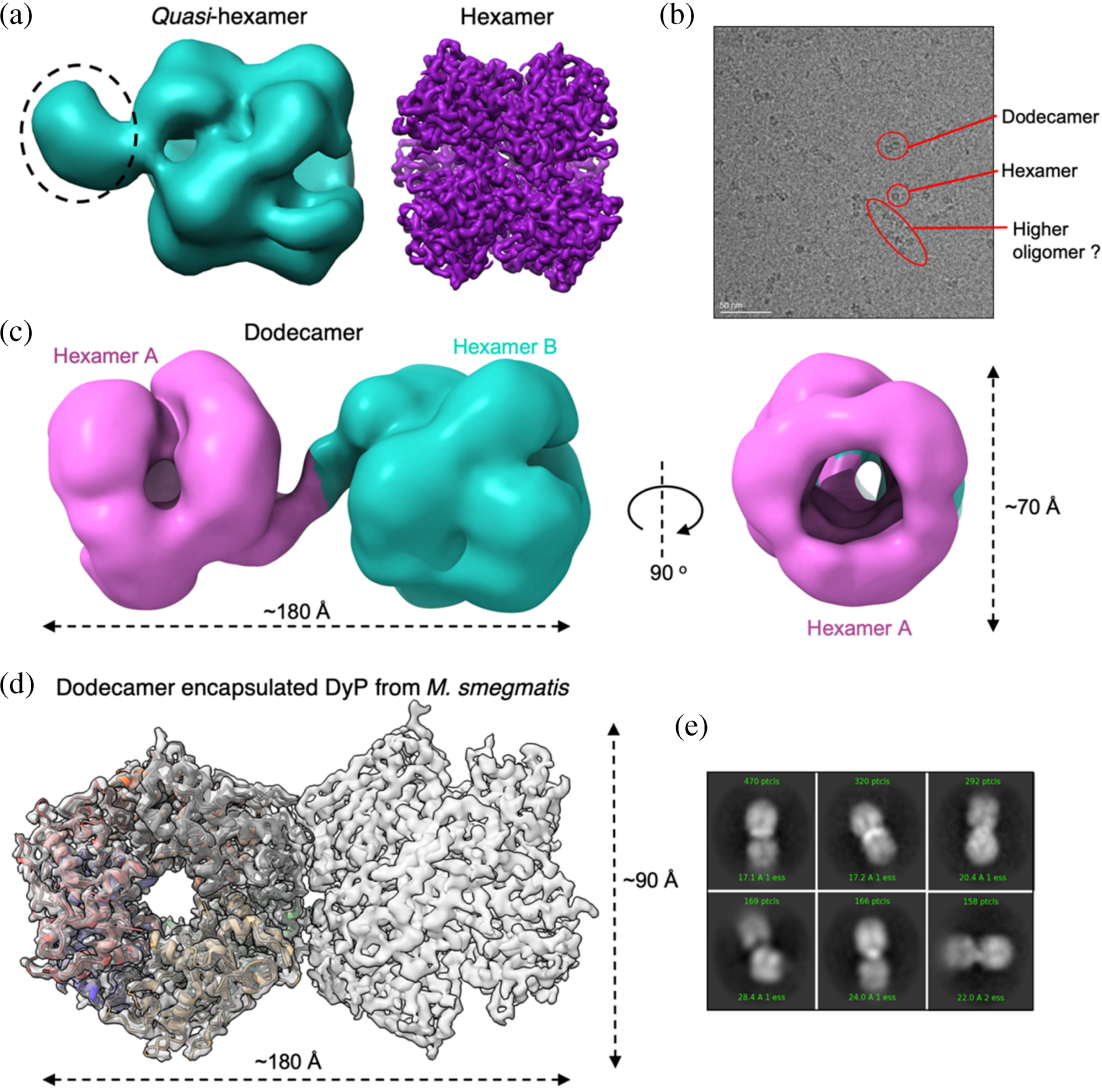
Higher‐order oligomer states of *Streptomyces lividans* DtpB. (a) Cryo‐EM map of the DtpB *quasi*‐hexamer (blue/green) with the additional density compared to the DtpB hexamer map (purple) shown as a dashed circle. (b) Example micrograph with red circles indicating three different oligomer states of DtpB. (c) Cryo‐EM maps and dimensions of the DtpB dodecamer. (d) Cryo‐EM map (3.7 Å resolution) for the dodecamer oligomer of the encapsulated dye‐decolorizing peroxidase from *Mycobacterium smegmatis* (PDB 7BOK) (Tang et al., 2021). (e) Different orientations of 2D classes of the dodecamer. DyP, dye‐decolorizing peroxidase.

Having identified the hexamer assembly as the dominant species in the single particle analysis, we next investigated whether other assembly states could be identified within our cryo‐EM datasets of DtpB (Figure S4). An example of a micrograph where at least two more higher‐order oligomers could be identified is shown in Figure 5b. From extensive 2D particle classification, two further cryo‐EM maps could be obtained (Figure S5), which comprise a hexamer assembly with an area of additional density (*quasi*‐hexamer) and an assembly comprising two hexamers, essentially creating a dodecamer (Figure 5e). The identification of a dodecamer from the single particle analysis is consistent with AUC data where the presence of a sedimentation species with a predicted dodecamer assembly was determined (Table 1). Due to only a small subset of particles corresponding to these two assemblies, we were only able to resolve low resolution cryo‐EM maps to 14.5 and 13.4 Å resolution for the *quasi*‐hexamer and the dodecamer, respectively (Figure S5). The low resolution of the maps makes it challenging to model structures with any atomic level precision. However, we were able to dock and orientate a DtpB hexamer into the map with extra density (Figure 5a). As to what this density protrusion arises from is not immediately apparent. However, it does appear to be important in the formation of the dodecamer assembly as an interaction between the protrusion on each hexamer occurs, assisting in forming the higher‐order oligomer with D3 symmetry retained (Figure 5c). A cryo‐EM structure for a dodecamer of a compartmentalized DtpB isolated in an encapsulin shell from *M. smegmatis* has been reported at 3.7 Å resolution (Tang et al., 2021) and is compared with our DtpB dodecamer (Figure 5d). For *M. smegmatis* the two hexamers are stacked against each other in a manner that eliminates the D3 symmetry (Figure 5d), with the dodecamer arrangement now best described as obeying D2 symmetry. This arrangement of hexamers could be enforced through the necessity to pack two hexamers into the confined space of an encapsulin nanoparticle lumen, whereas in our experiments and for DtpB in a cellular environment, no such restrictions are imposed. In either case, we note that access channels to and from the heme sites remain accessible.

## 3 DISCUSSION

For both A‐type DyPs, the sedimentation studies reveal a homodimer species dominates the solution landscape, with PISA analysis consistent with the homodimer observed in the crystallographic asymmetric unit being the likely functional state. In the case of DtpB, the sedimentation results demonstrate that a hexamer state dominates in solution, with the thermodynamic parameters obtained through PISA analysis again consistent with the hexamer observed in the crystallographic asymmetric unit and the dominant cryo‐EM species, being the most likely physiological assembly. Thus, a combination of AUC and structural analysis provides strong validation for the likely functional oligomer states. All DyPs react readily with H_2_O_2_ to form a Compound I species with considerable oxidizing potential (~1 V vs NHE). We have extensively studied the kinetic mechanism underpinning the formation of Compound I and subsequent reduction to Compound II in each of the *S. lividans* DyPs upon reaction with H_2_O_2_ (Chaplin et al., 2017; Lučić et al., 2022; Lučić, Chaplin, et al., 2020; Lučić, Svistunenko, et al., 2020), but have not observed any kinetic evidence to suggest that there is inequivalence in heme reactivity between the monomer units of the respective homomer, or even, allostery. This may be contrasted with Pupart et al. (Pupart, Vastšjonok, et al., 2024) who report using steady state assays with the DtpB from *S. coelicolor* that the catalytic activity has a dependence on the oligomeric state of the enzyme. This observation underpins the importance of the present work, as insights into all possible oligomeric states, and their structural makeup feeds into functional and mechanistic connotations. The remaining discussion is framed around the context of the functional role of *S. lividans* DyPs as homomers and seeks to integrate a wealth of mechanistic insight gained from our previous work (Chaplin et al., 2017, 2019; Lučić et al., 2021, 2022; Lučić, Chaplin, et al., 2020; Lučić, Svistunenko, et al., 2020).

**FIGURE 6:**
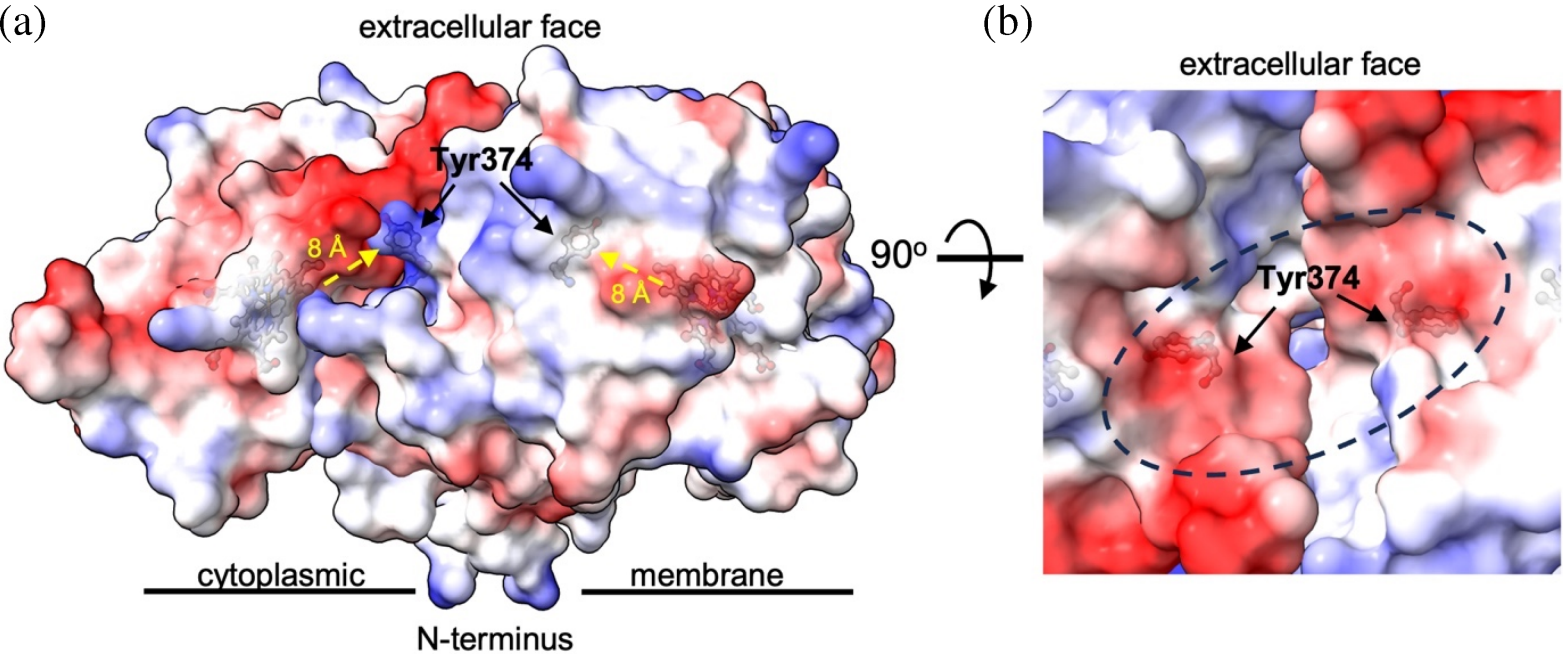
Mechanism of substrate oxidation by the DtpA homodimer. (a) Electrostatic surface map with the truncated N‐terminus (residues Δ1–69) orientated toward the cytoplasmic membrane. The heme and Tyr374 are represented in sticks with the yellow arrows indicating the distance the electron moves from the heme to form a tyrosyl radical. (b) View looking down the C2 symmetry axis of the homodimer. The dashed oval illustrates the putative substrate binding site created on forming a homodimer, with the Tyr374 residue visible beneath.

The physiological substrates for DyPs, particularly bacterial members are unknown. However, in vitro substrates, such as anthraquinone dyes are large bulky molecules which make access to the heme pocket challenging if a proximity to the heme is required for oxidation to occur. To circumvent this issue, several studies with A and C/D‐type DyPs have revealed that substrate oxidation proceeds via long range electron‐transfer (ET) pathways consisting of chains of ET active residues, for example, Tyr, Trp, Cys, and Met that serve to rapidly dissipate the strongly oxidizing equivalents stored on the heme to a site near or on the protein surface, where the oxidizing equivalent can be quenched (reduced) by a substrate (Baratto et al., 2015; Chaplin et al., 2019; Linde et al., 2015). For DtpA we have previously identified an ET pathway from the heme, which in the absence of a substrate, culminates in the formation of a tyrosyl radical on Tyr374, that is located ~8 Å from the heme (Figure 6a). On replacing the Tyr with a Phe, a 200‐fold decrease in the oxidation rate of a substrate was observed, leading to the proposal that Tyr374 was part of an efficient ET pathway to facilitate rapid substrate oxidation (Chaplin et al., 2019).

As the functional assembly of DtpA is here confirmed as a homodimer, how does this oligomer state influence the previous mechanistic proposal of substrate oxidation via Tyr374? DtpA is predicted to possess a transmembrane N‐terminal helix and would therefore be anchored to the cytoplasmic membrane. If we orientate the DtpA homodimer as shown in Figure 6a, with the truncated N‐terminus pointing toward the cytoplasmic membrane, Tyr374 which is located on the distal side of the heme (the side to which H_2_O_2_ binds) is found to be located well away from the membrane side. Furthermore, the previously identified H_2_O_2_ access tunnels leading from the protein surface to the heme are unobstructed in the homodimer (Chaplin et al., 2019). On forming the homodimer, a well‐defined surface cavity is created either side of the twofold symmetry axis that is dominated by two negative surface charge patches (Figure 6b). Tyr374 is found to lie directly beneath these negative patches with a distance to the surface of ~5 Å. We also note that this cavity would be capable of accommodating a symmetrical substrate molecule. Thus, the DtpA homodimer is clearly well optimized for long range ET from the heme via the distal Tyr374, and then to the surface cavity formed on creation of the twofold symmetry axis of the homodimer, with the distinct possibility of two electrons being delivered simultaneously to a symmetrical substrate positioned in the surface cavity. Such a possibility may help explain the complex kinetic behavior reported among several A‐type DyPs (Chen et al., 2015; Shrestha et al., 2016), with a recent study highlighting that two‐electron reductants display no cooperativity, whereas one‐electron reductants show positive cooperativity in steady‐state assays (Pupart, Lukk, et al., 2024). Notably, a negatively charged surface cavity also forms on creation of the twofold symmetry axis in the DtpAa oligomer. However, we have recently demonstrated that in the absence of a Tyr residue at the homologous 374 position in DtpAa, no long range ET from the heme via the distal pathway occurs (Lučić et al., 2023), corroborating the extremely low reactivity of DtpAa with many organic substrates (Lučić, Chaplin, et al., 2020). Thus, despite the two homodimers possessing a similar putative substrate binding site formed along the twofold symmetry axis of the oligomer, the absence of a viable ET pathway in DtpAa may account for the distinct differences in substrate reactivity between these two A‐type homologs (Chaplin et al., 2017; Lučić, Chaplin, et al., 2020).

**FIGURE 7:**
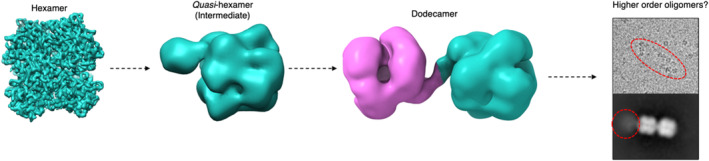
A putative assembly pathway for the formation of a dodecamer and higher‐order oligomers (red circle) of a B‐type dye‐decolorizing peroxidase.

A hexamer assembly is unequivocally determined to be the basic functional unit of DtpBs, which can exist free in the bacterial cytosol or when an encapsulin gene is present in the bacteria can become compartmentalized as cargo within the encapsulin lumen (Giessen & Silver, 2017). A cryo‐EM structure has illustrated that two DtpB hexamers from *M. smegmatis* can be compartmentalized in an encapsulin lumen to form a dodecamer assembly (Tang et al., 2021). Here, we have shown by AUC and cryo‐EM that *S. lividans* DtpB, has the inherent capability of forming a dodecamer oligomer and possibly even higher order assemblies (Figure 5b), albeit at low concentrations. Based on the single particle EM analysis a case can be made that in solution the pathway for dodecamer assembly is facilitated through an intermediate species, that is, the *quasi*‐hexamer (Figure 7). The low population of this and the subsequent dodecamer assembly may suggest that a co‐factor or binding partner that co‐purified at low levels with DtpB is responsible for interacting asymmetrically with each stable hexamer to form the dodecamer with D3 symmetry and with no interacting hexamer interfaces (Figure 5c). In contrast, the compartmentalized *M. smegmatis* DyP through being forced into the restricted lumen‐like space of an encapsulin forms a stable interface with several H‐bonding interactions between the two hexamers (Tang et al., 2021), promoting the view that compartmentalization plays a significant role in overcoming energy barriers for interaction and stabilizing higher‐order assembly states.

The structural, kinetic, and spectroscopic properties of DtpB, as well as other characterized DtpBs, set them apart from A‐type DyPs. This leads to several questions that need to be addressed when considering the present study, and in the wider context of their functional role, whether “free” in the cytosol or compartmentalized. Perhaps the most intriguing overall question is why have DtpBs evolved to react with H_2_O_2_ and form a Compound I which is so very long lived?

Unlike in DtpA, no long‐range ET pathways have been identified in DtpB, as evident from EPR spectroscopy through the absence of tyrosyl or tryptophanyl radicals following mixing with H_2_O_2_ (Lučić, Svistunenko, et al., 2020). Likewise, the absence of radical species in other DtpBs including those that are encapsulated following mixing with H_2_O_2_ is also noted (Roberts et al., 2011). An exception is the DtpB from *Klebsiella pneumoniae*, which a recent cryo‐EM structure reveals also to be a hexamer assembly when not encapsulated (Jones et al., 2024). EPR spectroscopy with the ferric heme “resting state” of *K. pneumoniae* DyP displays spectral features consistent with the presence of residual tyrosyl radical species. However, on addition of H_2_O_2_ to the ferric state, these residual resting state radical species do not increase in population, and neither do the appearance of new signals arising from other Tyr or Trp residues, that would indicate the potential for long range ET pathways (Nys et al., 2021; Pfanzagl et al., 2018). Furthermore, DtpBs generally exhibit low reactivity with the bulky synthetic dyes (often two‐electron donors), compared to more classical smaller peroxidase substrates (one electron‐donors) such as ABTS (Pfanzagl et al., 2018; Pupart, Vastšjonok, et al., 2024; Roberts et al., 2011; Shrestha et al., 2017). Although experimentally, long‐range ET pathways have yet to be detected in DtpBs, it does not mean they do not exist. Therefore, the lower reactivity in DtpBs for synthetic dyes may in part be explained by their inability to fit through the access channels identified in the hexamer assembly (Figure 4c) to come close to the heme, and in the absence of optimal long range ET pathways.

We have shown that DtpB is designed such that the acceptance of a single electron is unfavored by the fact that the heme pocket is “dry,” which determines that an electron can only be accepted by Compound I if accompanied by a proton, even though a proton is not required for reduction to Compound II (Lučić et al., 2022). This mechanistic arrangement essentially dictates that DtpB acts as a two‐electron acceptor, such that Compound I is reduced to Compound II and then the ferric state in a concerted manner (Lučić et al., 2022). Note that this is also the case for *M. tuberculosis* DyP which is an encapsulated DtpB (Lučić unpublished data). Therefore, this mechanistic behavior is difficult to make consistent with DtpBs acting in antioxidant defense as has been proposed in several reports characterizing the compartmentalization of DyPs (Contreras et al., 2014; Lien et al., 2021; Tang et al., 2021), as reaction of a single heme with H_2_O_2_ will form a long‐lived Compound I, while to act as a defense enzyme, DtpB, or any other DtpB would need to cycle to clear H_2_O_2_ from the environment.

A second question is, why if each heme acts independently as we have shown, do DtpBs form a hexamer and even higher‐order oligomers? A view promoted in the literature, particularly from a standpoint of encapsulation, is to provide a functional advantage through the increased concentration of heme in a localized space. However, the longevity of Compound I may suggest that DtpB and other DtpBs (including encapsulated ones) efficiently (and perhaps remarkably) safely store oxidizing equivalents in readiness for an interaction with a cognate substrate. This is in part effected by the enzyme acting as a two‐electron acceptor. Therefore, we propose that DtpBs whether “free” or compartmentalized act as way stations for the removal of oxidizing equivalents through a cellular substrate that is a two‐electron donor that is only temporally present. A role for compartmentalization could therefore be to direct the DyP cargo to a localized region of the cognate substrate leading to controlled access of the substrate into the capsid.

## 4 CONCLUSION

In conclusion, by reporting the first comprehensive AUC dataset for a set of DyPs from the same organism we reveal that A‐ and B‐type sub‐family members are unequivocally functional homomers. This continues our holistic approach to understanding DyP mechanism and function where we have been focused on elucidating the chemistry of the heme pocket associated with the reaction with H_2_O_2_, and the movement of electrons, with the results of this study allowing us to place in context some of these previous findings. The symmetry imposed through creating A‐type homodimers putatively allows in DtpA for the synchronous delivery of two electrons to a symmetry formed putative substrate binding site. Through applying cryo‐EM to DtpB, we have been able to obtain the highest resolution single particle structure of a DtpB to date, and most significantly have been able to detect and visualize higher‐order oligomers. We therefore not only verify our AUC data using a structural approach, but also illustrate that such species can exist in the absence of compartmentalization by an encapsulin. Whereas the existence of higher‐order oligomers is a challenging concept to understand, we propose that the DtpB hexamer assembly performs an insulating role in so much as it creates an enzyme that functions to store oxidizing equivalents generated through reaction with H_2_O_2_, and will only react with the “right” substrate, in a two‐electron manner. Finally, functional roles for the DyP family of peroxidases are still not really known, but this work, together with a recent other (Pupart, Vastšjonok, et al., 2024), highlights that oligomer states likely play a prevalent role.

## 5 MATERIALS AND METHODS

### 5.1 Over‐expression and purification of DyPs

A pET28a vector (Kan^r^) containing the DNA to overexpress the desired DyP from *S. lividans* was transformed into chemically competent *Escherichia coli* BL21(DE3) cells, with single transformants used to inoculate 10 mL LB pre‐cultures grown overnight at 37°C. The overnight cultures were then used to inoculate 2 L flasks containing 1.4 L of LB and cultured at 37°C with shaking at 180 rpm. At an OD_600_ of 0.8, the following supplements (final concentrations indicated) were added to the flasks; 100 μM iron citrate (Merck), 250 μM 5‐aminolevulinic acid (Merck) and 500 μM isopropyl β‐d‐1‐thiogalactopyranoside (Melford Chemicals). For DtpA and DtpAa, but not DtpB, CO gas was bubbled through the culture flasks before sealing. Following the supplement additions growth continued at 30°C with shaking at 100 rpm for 16 h. Cultures were harvested by centrifugation followed by resuspension of the cell pellets and lysis using an Emulsiflex (C5‐Avenstein). Subsequent chromatography purification steps for all DyPs were performed as previously reported (Chaplin et al., 2017; Lučić, Chaplin, et al., 2020; Lučić, Svistunenko, et al., 2020; Petrus et al., 2016).

### 5.2 Analytical ultracentrifugation

SV experiments were performed in a Beckman Optima XL‐I analytical ultracentrifuge equipped with scanning absorbance optics and an An50 Ti rotor. DyP samples were diluted into 50 mM HEPES pH 7.0, 100 mM NaCl to an absorbance of ~0.8 at 410 nm, to give final protein concentrations of between 0.75 and 0.85 μM. These were loaded into charcoal‐filled Epon double‐sector cells fitted with quartz windows. Reference sectors were filled with the buffer, and centrifugation was performed at 40,000 rpm at 20°C. Two hundred radial scans were recorded for each sample at 410 nm. Data were analyzed using the *c*(*S*) distribution model in SEDFIT (Schuck, 2000). The partial specific volume for each DyP was calculated from the amino acid sequence using the program SEDNTERP (Harding et al., 1992). The molecular weight and S_20,W_ for each species was calculated using SEDFIT (Schuck, 2000).

### 5.3 Cryo‐EM grid preparation

Aliquots of DtpB (3 μL of ~6 mg/mL) were mixed with 8 mM CHAPSO (final concentration, Sigma) before being applied to Holey Carbon grids (Quantifoil Cu R1.2/1.3, 300 mesh), glow discharged for 60 s at a current of 25 mA in a PELCO Easiglow (Ted Pella, Inc). The grids were then blotted with filter paper once to remove any excess sample, and plunge‐frozen in liquid ethane using a FEI Vitrobot Mark IV (ThermoFisher Scientific Ltd) at 4°C and 95% humidity.

### 5.4 Cryo‐EM data acquisition and image processing

Cryo‐EM data were collected on either a Titan Krios (hexameric DtpB structure) or a Talos Arctica (dodecamer and *quasi*‐hexamer DtpB structures) with data collection parameters reported in Table 3. For the hexameric DtpB structure, 1400 movies were collected, and for the dodecamer and *quasi*‐hexamer DtpB structures 379 movies were collected, in accurate hole centering mode using EPU software (ThermoFisher). CTF correction, motion correction, and particle picking were performed using Warp (Tegunov & Cramer, 2019). For the hexamer DtpB structure, 71,074 particles were picked by boxnet2 masked neural network model in Warp and were imported to CryoSPARC (Punjani, Brubaker, et al., 2017; Punjani, Rubinstein, et al., 2017) for all subsequent processing. These particles were initially subjected to two‐dimensional (2D) classification, and produced clear images of hexamers and dodecamers. Particles corresponding to different classes (either hexamer or dodecamer) were selected and optimized through multiple iterative rounds of heterogeneous refinement as implemented in CryoSPARC (Punjani, Brubaker, et al., 2017; Punjani, Rubinstein, et al., 2017). Particles not containing the DtpB hexamer or dodecamer were discarded. The best models were then further refined using homogenous refinement and finally non‐uniform refinement in CryoSPARC (Punjani, Brubaker, et al., 2017; Punjani, Rubinstein, et al., 2017). 24,402 particles selected from these classes were used to generate a hexamer structure of DtpB with D3 symmetry imposed (Figure S2).

For the dodecamer and *quasi*‐hexamer, 43,144 particles were picked by boxnet2 masked neural network model in Warp (Tegunov & Cramer, 2019) and imported to CryoSPAC (Punjani, Brubaker, et al., 2017; Punjani, Rubinstein, et al., 2017) for subsequent processing. These particles were initially subjected to 2D classification and produced clear images of *quasi*‐hexamers and dodecamers (Figure S5). Particles corresponding to these different classes were selected and optimized through multiple iterative rounds of heterogeneous refinement as implemented in CryoSPARC (Punjani, Brubaker, et al., 2017; Punjani, Rubinstein, et al., 2017). Particles not containing DtpB were discarded. The best models were then further refined using homogenous refinement and finally non‐uniform refinement in CryoSPARC (Punjani, Brubaker, et al., 2017; Punjani, Rubinstein, et al., 2017). 3964 particles selected from these classes were used to generate a dodecamer structure of DtpB with C1 symmetry imposed. 5324 particles selected were used to generate a *quasi*‐hexamer structure. This classification process is summarized schematically in Figure S5. The final reconstructions obtained had overall resolutions (Table 3), which were calculated by Fourier shell correlation at 0.143 cut‐off (Figures S1 and S4).

### 5.5 Structure refinement and model building

The X‐ray crystal structure of DtpB (PDB ID: 6YR4) was used as an initial template and rigid‐body fitted into the cryo‐EM density for the hexamer, *quasi*‐hexamer and dodecamer in UCSF chimera (Pettersen et al., 2004) and manually adjusted and rebuilt in Coot (Emsley et al., 2010). Namdinator (Kidmose et al., 2019) was used to adjust the final structures and several rounds of real space refinement were then performed in PHENIX (Afonine et al., 2018) before final validation and deposition to the PDB and EMDB for the hexamer map and structure (Table 3). Maps for the *quasi*‐hexamer and dodecamer were not deposited due to their low resolutions.

## AUTHOR CONTRIBUTIONS

**Marina Lučić:** Conceptualization; investigation; formal analysis. **Thomas Allport:** Investigation; formal analysis; data curation. **Thomas A. Clarke:** Investigation; formal analysis; writing – review and editing. **Lewis J. Williams:** Investigation. **Michael T. Wilson:** Conceptualization; writing – review and editing. **Amanda K. Chaplin:** Conceptualization; investigation; writing – review and editing; funding acquisition; formal analysis; resources; supervision; data curation. **Jonathan A. R. Worrall:** Conceptualization; funding acquisition; writing – original draft; writing – review and editing; formal analysis; resources; supervision.

## CONFLICT OF INTEREST STATEMENT

There are no conflicts of interest to declare.

## Supporting information


**Data S1.** Supporting information.
