# Supplementary material for: The oligomeric states of dye‐decolorizing peroxidases from Streptomyces lividans and their implications for mechanism of substrate oxidation
Source: Protein Sci. 2024 Jun 12;33(7):e5073. doi: 10.1002/pro.5073 (PMC11168072; doi:10.1002/pro.5073)
Supplement: Supplementary file 1 — Data S1. Supporting information. [file PRO-33-e5073-s001.docx]

**Supporting Information**

**The oligomeric states of dye-decolorizing peroxidases from *Streptomyces lividans* and their implications for mechanism of substrate oxidation**

Marina Lučić,^1^ Thomas Allport,^2^ Tom A. Clarke,^3^ Lewis J. Williams,^1^ Michael T. Wilson,^1^ Amanda K. Chaplin,^2^* Jonathan A.R. Worrall^1^*

^1^School of Life Sciences, University of Essex, Wivenhoe Park, Colchester, CO4 3SQ U.K. ^2^Leicester Institute for Structural and Chemical Biology, Department of Molecular and Cell Biology, University of Leicester, Leicester, LE1 7RH U.K. ^3^School of Biological Sciences, University of East Anglia, Norwich Research Park, Norwich, NR4 7TJ U.K.

*To whom correspondence should be addressed: jworrall@essex.ac.uk; ac853@leicester.ac.uk


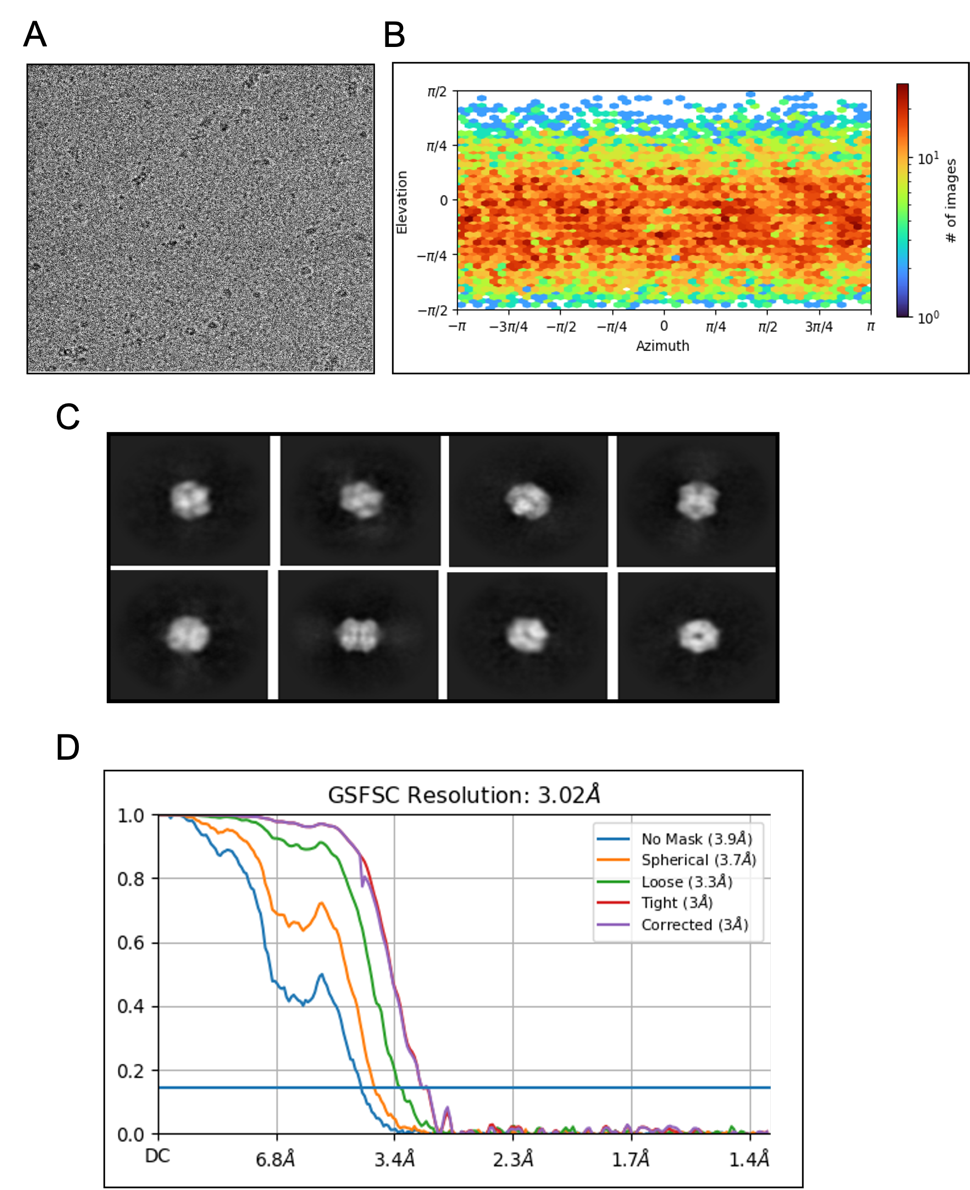


**Figure S1:** Cryo-EM data of *S. lividans* DtpB hexamer. A) Example micrograph with DtpB protein particles. B) Heat map displaying the number of particles for each viewing angle calculated in) CryoSPARC (1; 2). C) Different orientations of 2D classes of the DtpB hexamer. D) FSC curves illustrating an overall resolution of 3.02 Å.


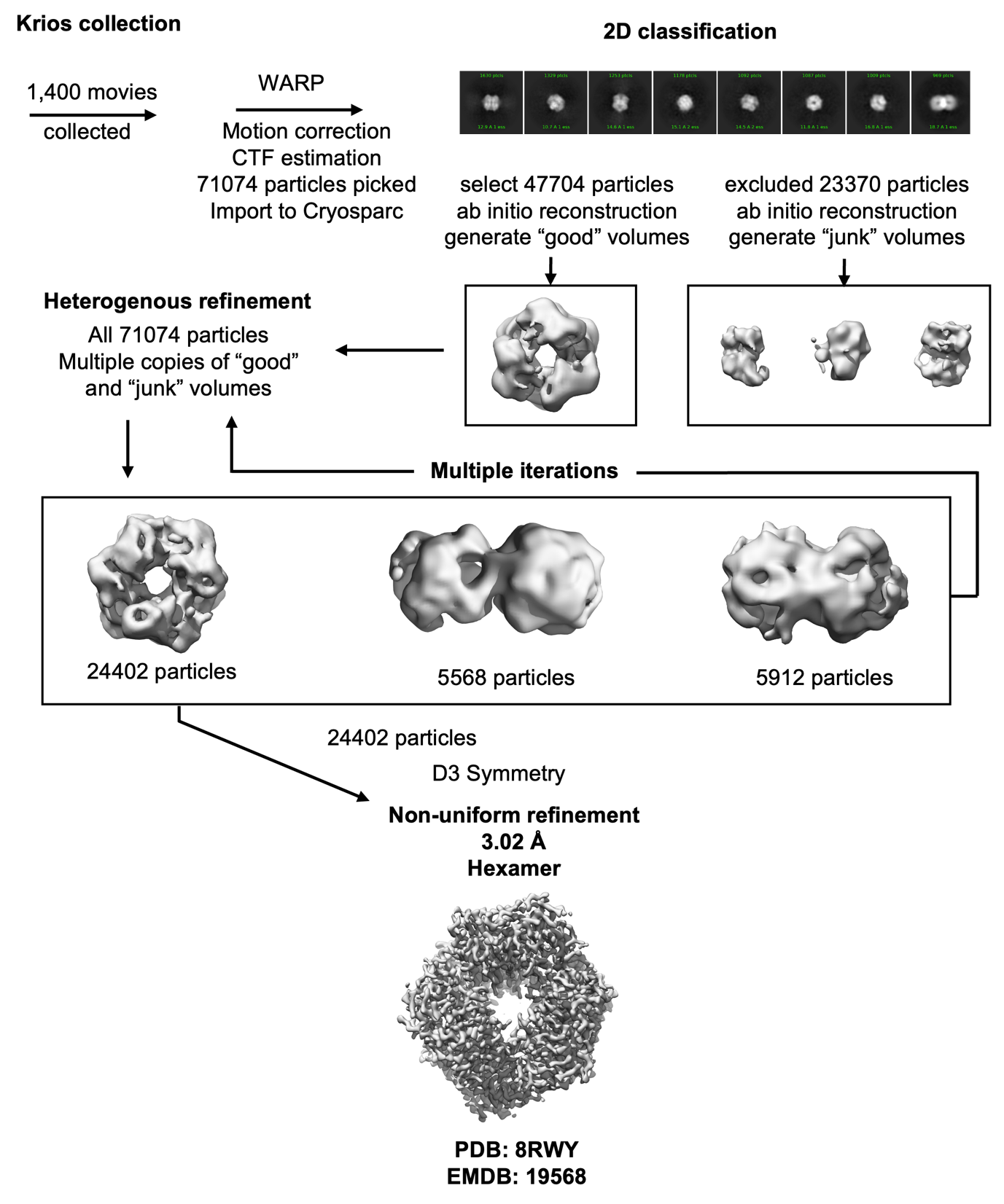


**Figure S2:** Cryo-EM data processing workflow for DtpB hexamer from *S. lividans*.


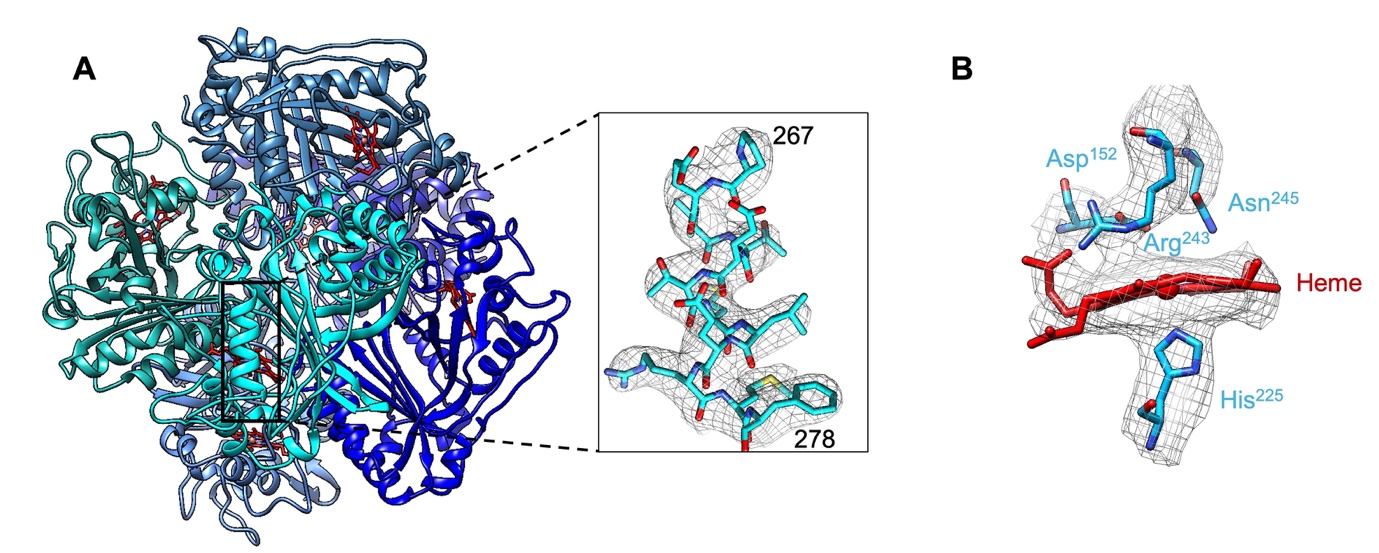


**Figure S3:** Cryo-EM structure of DtpB determined to 3.02 Å resolution. A) Representative cryo-EM density (grey mesh) for a helix region (residues 267-278 shown in sticks) within the hexamer assembly. B) Illustrative cryo-EM density (grey mesh) for the heme environment, with heme and residues shown in sticks.


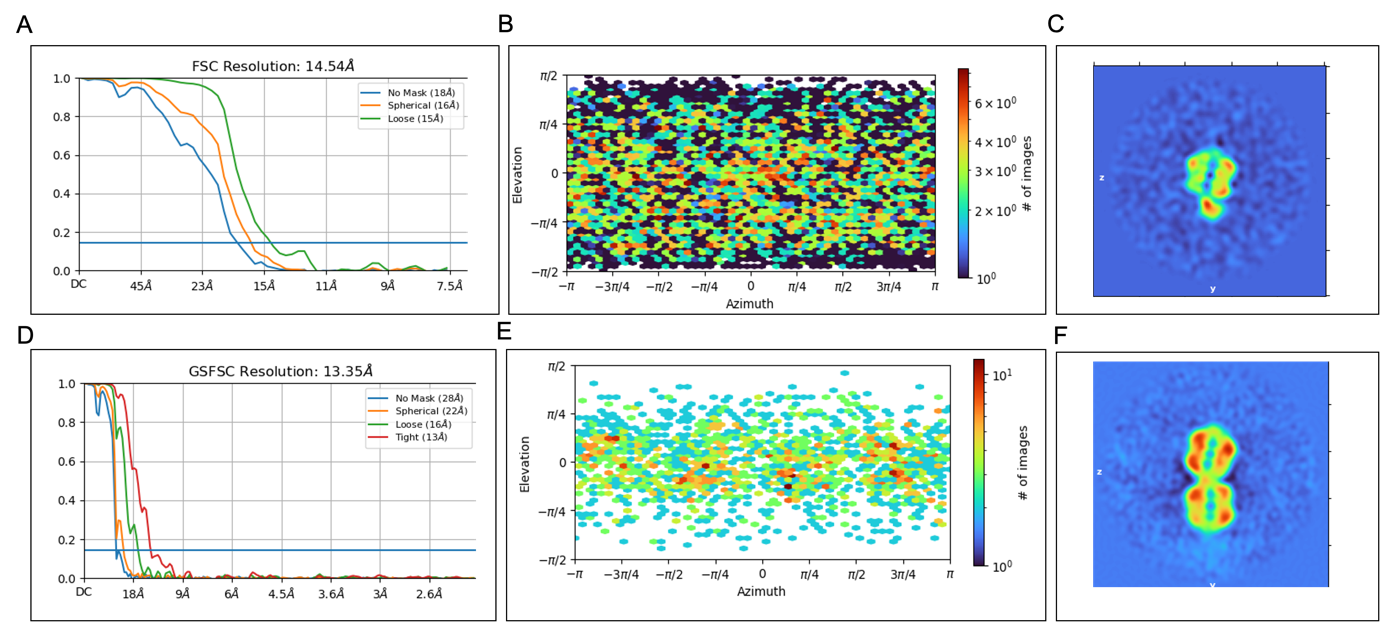


**Figure S4:** Cryo-EM data of the *quasi*-hexamer and dodecamer of DtpB. A) FSC curve illustrating an overall resolution of 14.54 Å for the *quasi*-hexamer. B) Heat map displaying the number of particles for each viewing angle calculated in CryoSPARC (1; 2). C) Example 3D reconstruction image of the *quasi*-hexamer from CryoSPARC (1; 2). D) FSC curves illustrating an overall resolution of 13.35 Å for the dodecamer. E) Cryo-EM maps and dimensions of the dodecamer. F) Example 3D reconstruction image of the dodecamer from CryoSPARC (1; 2).


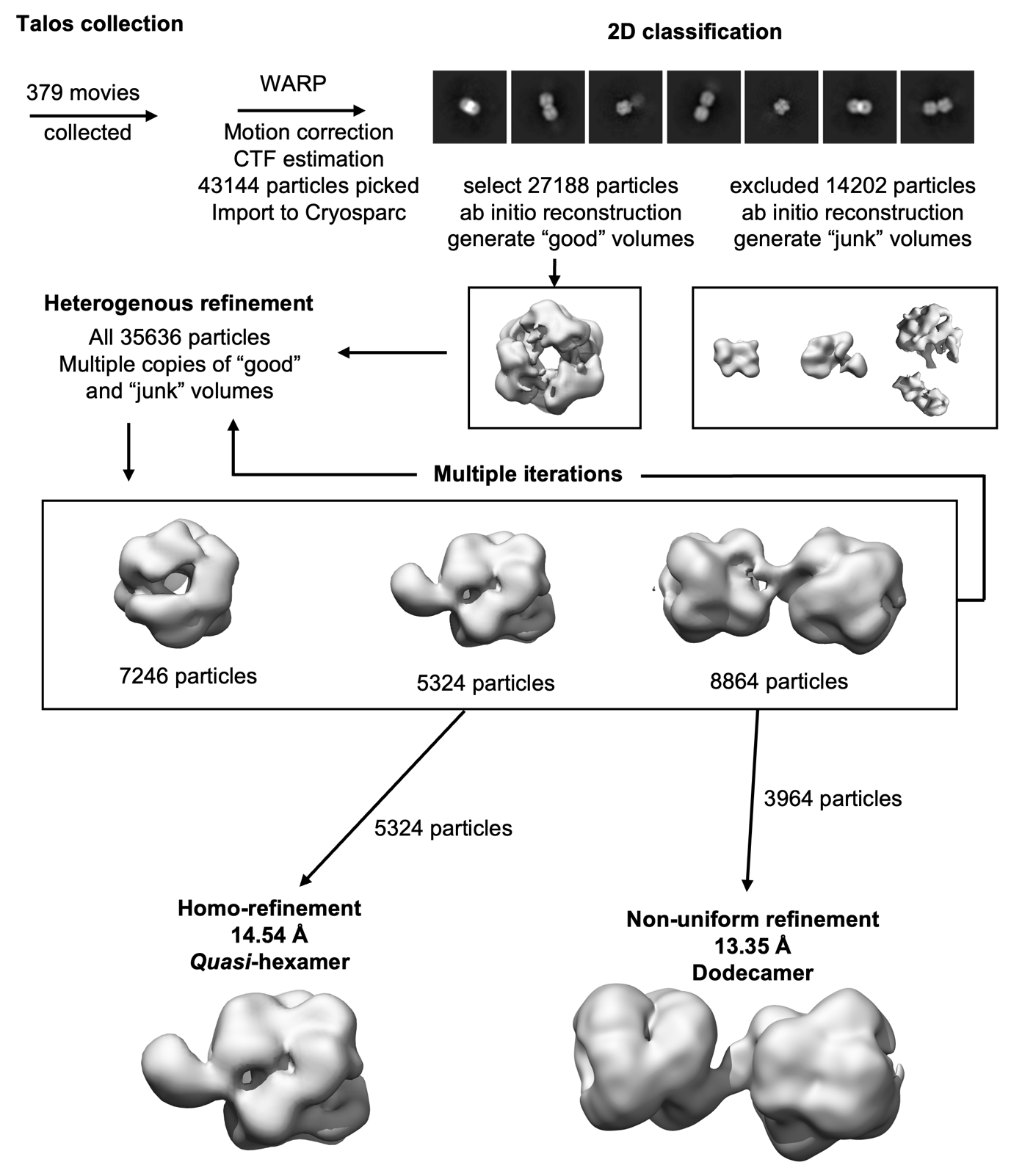


**Figure S5:** Cryo-EM data processing workflow for DtpB *quasi*-hexamer and dodecamer from *S. lividans*.

**REFERENCES**

1. Punjani A, Brubaker MA, Fleet DJ (2017) Building Proteins in a Day: Efficient 3D Molecular Structure Estimation with Electron Cryomicroscopy. IEEE Trans Pattern Anal Mach Intell 39:706-718.

2. Punjani A, Rubinstein JL, Fleet DJ, Brubaker MA (2017) cryoSPARC: algorithms for rapid unsupervised cryo-EM structure determination. Nat Methods 14:290-296.
